# Production responses of dairy cows to precision feeding based on historical performance during short-term changes in supplementation

**DOI:** 10.1093/jas/skaf317

**Published:** 2025-09-15

**Authors:** Vinícius C. Souza, Claire B. Gleason, Tanner P. Price, Bárbara R. dos Reis, Sathya Sujani, Ty C. Davis, Douglas M. Liebe, Kristy M. Daniels, Robin R. White

**Affiliations:** School of Animal Sciences, Virginia Tech, Blacksburg, VA 24061; Present address: Department of Animal Science, University of São Paulo, Pirassununga, SP 13635-900, Brazil; School of Animal Sciences, Virginia Tech, Blacksburg, VA 24061; School of Animal Sciences, Virginia Tech, Blacksburg, VA 24061; School of Animal Sciences, Virginia Tech, Blacksburg, VA 24061; School of Animal Sciences, Virginia Tech, Blacksburg, VA 24061; School of Animal Sciences, Virginia Tech, Blacksburg, VA 24061; School of Animal Sciences, Virginia Tech, Blacksburg, VA 24061; School of Animal Sciences, Virginia Tech, Blacksburg, VA 24061; School of Animal Sciences, Virginia Tech, Blacksburg, VA 24061

**Keywords:** algorithm, feed efficiency, milk yield, precision feeding

## Abstract

The increasing automation of dairy systems enables precision feeding, where dietary supplements can be tailored to individual animals. However, data describing short-term production responses to such supplementation are lacking, limiting the development of individualized feeding algorithms. This study aimed to 1) examine dairy cow responses to short-term changes in top dress supplementation and 2) evaluate two simple precision feeding strategies compared to a conventional total mixed ration (TMR). Twenty-four lactating Holstein cows were assigned to one of four top-dress treatments: soybean meal (SBM), corn grain (CG), corn gluten feed (GLT), or no supplement in a replicated 4 × 4 Latin square during a 36-d training phase. Subsequently, cows were reassigned to three feeding groups: Control (conventional TMR), algorithm 1 (based on mean past performance), or algorithm 2 (based on the slope of past responses), and evaluated over four 7-d testing periods. During training, dry matter intake (DMI) and milk yield (MY) increased on all top dress treatments relative to the Control treatment (*P *< 0.001), but feed efficiency (FE; MY/DMI) declined (*P *< 0.001). SBM reduced milk fat percentage and yield (*P *< 0.05), while milk protein, body weight (BW), and energy-corrected milk (ECM) were unaffected (*P *> 0.27). During testing, DMI tended to increase in Algorithm 1 vs. Control (25.2 vs. 22.7 kg/d; *P *= 0.08), with a corresponding tendency for reduced FE (1.45 vs. 1.61; *P *= 0.07). No performance differences were detected between algorithm 2 and Control (*P *> 0.50). Milk composition, ECM, and BW were similar across feeding groups (*P *> 0.33). Feed cost, milk revenue, and income over feed cost (IOFC) did not differ between precision-fed and control groups (*P *> 0.33). These findings suggest that short-term historical production responses may be insufficient to inform future supplementation decisions and that more advanced approaches, including new data streams, may be necessary to improve individual cow performance using precision feeding strategies.

## Teaser

A simple precision feeding strategy did not change feed costs, milk revenue, or milk revenue over feed costs compared with total mixed ration feeding. The lack of performance benefit from employing this simple precision feeding approach (based on animal historical performance) indicates that past animal performance responses to nutritional supplementation may not be reliable indicators of future performance behavior, even across short forecasting windows.

## Introduction

With the ever-increasing adoption of precision technologies in the livestock industry, managing animals at the individual level is rapidly becoming more feasible. Many examples of precision technologies are now commonly employed on dairy farms to monitor cow behavior and production parameters, such as pedometers (Roelofs et al., 2005; Mayo et al., 2019), eating and rumination monitors (Beauchemin, 2018), and in-parlor milk meters (Andreen et al., 2020). Behavioral and performance data collected by precision technologies can theoretically be utilized to more accurately estimate individual cow daily nutrient requirements (da Rosa Righi et al., 2020). The automation of milking and feeding procedures provides an opportunity to offer individual cows a feed supplement separate from the group ration they consume at the feed bunk. Leveraging individual cow nutrient requirement information along with an automated feeding system capable of delivering an individualized amount and type of supplement rather than the common type of supplement currently delivered by most automatic milking systems provides an opportunity for precision feeding. This approach has the potential to increase feed efficiency (FE) or milk yield (MY), thereby yielding economic benefits for producers and addressing concerns about environmental impact (Connor, 2015). However, such outcomes will depend greatly on how individualized strategies are implemented and evaluated. Previous feeding studies determined that cow performance parameters, including DMI, MY, FE, and certain milk components, will respond to immediate and short-term changes in the provision of individually-fed supplements with a considerable degree of variation between individual animals (Price et al., 2021). It is therefore feasible to expect that algorithms intended to formulate custom rations at the individual animal level need to be developed using individual animal data rather than group means.

Although diets are typically formulated to meet the requirements of the average cow within a group (NASEM, 2021), individual cows may still experience nutrient deficits or surpluses due to variability in nutrient use efficiency and intake, even when grouping strategies are adopted on dairy farms. This variation means that some cows may respond positively to additional nutrients, while others may not benefit or could even be oversupplied. The objective of precision or individualized feeding is therefore to tailor the nutrient supply to each cow’s specific needs or responses, rather than increasing concentrate feeding uniformly (Souza et al., 2022). Within this context, evaluating responses to incremental supplementation beyond group-formulated requirements provides a way to reveal heterogeneity in animal performance, which is essential for developing strategies to better match nutrient supply with individual requirements. Souza et al. (2022) explored this approach, fitting and testing precision feeding algorithms based on the individual animal data reported in Price et al. (2021); however, the resulting algorithms tended to target short-term intake depression to increase FE. While cows in this situation may maintain milk production levels by switching to body reserves in the short-term, the negative effects of prolonged intake depression on animal health and performance make this an undesirable feeding strategy. Precision feeding algorithms intended to optimize performance must therefore be carefully specified so as not to select supplement options that could lead to harmful or counterproductive downstream effects.

To build on this previous work, address the concerns therein, and further explore the development of precision feeding strategies, the present study sought to examine the individual production responses of mid-lactation cows to dietary changes and leverage these individual responses to train and test 2 precision feeding algorithms adjusted to avoid selecting for depressed DMI. The objectives were therefore to 1) measure the effect of feed supplements provided to individual cows on their DMI, MY, FE, and milk component concentrations, and 2) evaluate the abilities of two precision feeding strategies to optimize cow performance through the provision of supplements compared to the performance of cows receiving the base diet only. We hypothesized that 1) cows would respond to short-term changes in diet by altering productive output in a repeatable manner over the timeframe of the study, and 2) that cows fed using the precision feeding approaches would demonstrate an increase in performance compared to traditionally fed cows.

## Materials and Methods

### Animals, experimental design, and diets

All procedures involving animals were approved by the Institutional Animal Care and Use Committee of Virginia Tech (protocol #18–002). This study was conducted from September 24, 2019 to December 17, 2019. The animal subject criteria, experimental design, and procedures in this study closely follow those described in Price et al. (2021) and Souza et al. (2022); however, the present work was a separate experiment from that described in Price et al. (2021) and Souza et al. (2022), using the same experimental design and procedures, but different animals, base TMR, and feedstuffs. The study subjects consisted of 24 lactating Holstein cows (12 primiparous and 12 multiparous; 669 ± 60 kg BW; and 129 ± 16 DIM at the beginning of the study) housed in a free-stall barn pen fitted with a Calan Broadbent Feeding System (American Calan Inc.). Cows were monitored daily by research personnel and farm staff for signs of illness, injury, or significant weight loss, which would indicate a cow should be removed from the study. No cows required removal for these reasons. Cows were fed once daily at approximately 1300 h. Prior to study commencement, cows were given an acclimation period of 14 d to learn how to access their respective Calan doors. The study was divided into two phases: training and testing. The purpose of the training phase was to examine the short-term productive responses of cows to changes in diet and to utilize these data to train the precision feeding approaches (described below under *Feeding Algorithms*). The purpose of the testing phase was to evaluate the ability of different feeding approaches to optimize cow performance.

#### Dietary treatments

The diets in this work were designed to compare traditional feeding approaches, such as total mixed rations (TMR), to a flexible precision feeding strategy where a small portion of the diet is customized based on individual animal performance on a weekly basis. This strategy was used to represent an application of precision feeding that might leverage traditional alley-fed TMR allocation, with an individual feeder or a parlor feed allocation customized to individual cows. These examples were of interest because they allow more precise customization of feed allocated to individual cows without requiring major infrastructure changes on farms. Throughout the work, we refer to the TMR as either the TMR or the base of the ration, and to the supplemental feeds provided on an individual basis as the top dress.

The TMR used for all diets consisted mainly of corn and millet silages, corn grain, brewers’ grain, and soy hulls, and reflects the TMR fed to mid and late lactation cows at the Virginia Tech research dairy during the time of this study (Table 1). Top dress treatments included: solvent-extracted soybean meal (SBM), corn grain plus SBM (CG + SBM), or corn gluten feed plus SBM (GLT + SBM), or no top dress (Control). These treatments resulted in four diets: cows in the Control group received only the TMR, while cows in the other groups received the TMR plus their allocated top dress treatment. Feed supplements used for the top dress treatments leveraged commodity feeds commonly available on dairies. The formulated base and treatment rations are summarized in Table 2. In our previous work (Price et al., 2021; Souza et al., 2022), the amount of TMR and supplemental feed allocated to each animal was based on the substitution of 20% of the NEl of the diet; however, day-to-day feed and individual animal variation make this proportional substitution challenging to implement in practice. As such, in this iteration of the feeding experiment, we formulated diets using Agricultural Modeling and Training Systems Cattle Pro software (AMTS, LLC, Groton, NY; Table 2) to target a constant percent inclusion for each top dress. The TMR diet was formulated and checked against the animal requirements during the course of the experiment (NRC, 2001) to ensure requirements were met. The top dress inclusion percentages were formulated to maintain a similar calculated NEl content of the diets, while allowing different composition of other nutrients, predominantly RDP, to vary. The calculated RDP concentrations (% DM) for the SBM, CG + SBM, GLT + SBM, and Control diets were estimated at 10.3, 8.8, 9.3, and 8.3%, respectively (Table 2). Because different ingredients were used to generate these variable RDP levels, other trade-offs in nutrients also occurred with these diets, including shifts in the NDF and NFC contents compared with the control treatment (Table 2). The emphasis on RDP was driven by our previous precision feeding experiment, in which cows appeared to be most responsive to shifts in RDP (Price et al., 2021), rather than shifts in energy availability or changes in other nutrient contents. The four different diets (Control or TMR plus top-dress) evaluated in this study were designed to achieve a similar total daily dietary metabolizable energy intake (57.5 to 58.3 Mcal/d). The mean apparent dietary chemical composition across cow days was calculated and reported along with the standard deviation of apparent composition as the best available representation of diet chemical composition consumed by cows on each treatment. These values are summarized in Table 3.

**Table 1.** Ingredient composition of the base TMR

Ingredient	Inclusion percentage, DM basis
**Corn silage, brown midrib**	40.4
**Corn grain, dry, ground**	3.47
**Millet silage**	9.60
**Cottonseed, whole, with lint**	6.67
**Brewers grains, wet**	8.81
**Milk cow concentrate:**	31.0
**Soybean hulls, ground**	9.54
**Canola meal**	7.01
**Amino plus** ^1^	6.83
**Palmit 80** ^2^	1.50
**Blood meal, dried**	1.36
**Sodium bicarbonate**	0.87
**Limestone, ground**	0.87
**Potassium carbonate**	0.75
**Salt, white**	0.50
**Volclay 90** ^3^	0.30
**Molasses, cane**	0.23
**OmniGen-AF** ^4^	0.30
**Potassium magnesium sulfate**	0.16
**MHA, dry** ^5^	0.14
**Calcium phosphate, mono-dicalcium phosphate**	0.12
**Mepron** ^6^	0.10
**Diamond XPC** ^7^	0.070
**Selenium yeast, 0.06%**	0.070
**Ultrasorb** ^8^	0.052
**Clarifly** ^9^	0.052
**Biotin** ^10^	0.052
**Zinpro 5** ^11^	0.035
**Trace mineral blend**	0.035
**Vitamin A, D, E blend**	0.017
**Vitamin E premix, 60%**	0.017
**Rumensin 90** ^12^	0.0052
**Total**	100.0

1 Rumen protected soybean meal; Ag Processing, Inc.

2 Vegetable palm oil; rumen bypass fat; ADM Animal Nutrition.

3 Granular sodium bentonite; binder and digestive aid; American Colloid Company.

4 Immune support supplement; microbial ingredients, vitamins, and aluminosilicates; Phibro Animal Health Corporation.

5 Granular formulation of ALIMET; 84% methionine activity and 100% absorbed; NOVUS.

6 Rumen protected DL-methionine; RP Nutrients, Inc.

7 *Saccharomyces cerevisiae* fermentation product; Diamond V.

8 Mycotoxin deactivator; Micron Bio-Systems Ltd.

9 Diflubenzuron feed-through fly control; Central Garden and Pet Company—Central Life Sciences.

10 2,200 mg Biotin/kg.

11 Trace mineral concentrate containing 30,688.51 mg/kg manganese, 5,370.57 mg/kg copper, 306.30 mg/kg iron, 75,187.97 mg/kg zinc, 613.32 mg/kg iodine, and 1,687.43 mg/kg cobalt (DM basis); Zinpro Performance Minerals.

12 Monensin sodium energy supplement for increased milk production efficiency; Elanco Animal Health.

**Table 2.** Calculated composition of treatment rations

Ingredient DM, kg/d	Treatments			
	TMR	CG + SBM	GLT + SBM	SBM
**Corn silage**	9.36	8.45	8.45	8.45
**Dry ground corn**	0.83	2.04	0.75	0.75
**Millet silage**	2.23	2.00	2.00	2.00
**Whole cottonseed**	1.55	1.40	1.40	1.40
**Wet brewers grains**	2.05	1.85	1.85	1.85
**Milk cow concentrate** ^1^	7.19	6.50	6.50	6.50
**Corn gluten feed**	0.00	0.00	1.30	0.00
**Soybean meal**	0.00	0.74	0.74	2.05
**Calculated composition** ^2^				
**DM, %**	44.6	46.7	49.7	46.7
**CP, %**	15.5	16.3	17.2	18.7
**RDP, % DM**	8.25	8.77	9.29	10.3
**RUP, % CP**	46.9	46.2	46.1	45.0
**NDF, %**	38.7	36.6	37.5	36.1
**ADF, %**	24.0	22.5	22.7	22.4
**ADL, %**	10.1	9.9	9.8	10.0
**NFC, %**	33.8	35.5	33.3	33.3
**ME, Mcal/kg**	2.51	2.55	2.51	2.53
**NEl, Mcal/kg**	1.63	1.63	1.61	1.63
**NEm, Mcal/kg**	1.63	1.65	1.61	1.65
**Expected DMI, kg/d**	23.2	22.9	23.0	23.0
**ME allowable milk, kg/d**	37.0	37.0	36.0	36.6
**MP allowable milk, kg/d**	36.3	38.3	39.4	42.6

1 Milk cow concentrate is defined in Table 1.

2 Calculated values using the AMTS Cattle Professional software.

**Table 3.** Mean apparent composition of diets consumed based on proximate analysis of feed and orts

Nutrients	Apparent diet composition^1^			
	TMR^2^	CG + SBM^2^	GLT + SBM^2^	SBM^2^
**DM**	47.4 (5.0)	49.9 (1.6)	49.8 (1.5)	47.1 (1.8)
**CP**	16.7 (1.8)	16.7 (0.72)	17.0 (0.88)	17.3 (0.70)
**NDF**	35.8 (1.3)	33.5 (0.65)	33.4 (0.68)	33.8 (0.66)
**ADF**	19.4 (1.44)	18.0 (0.55)	18.0 (0.76)	18.4 (0.98)
**NFC** ^3^	36.7 (2.3)	35.4 (1.1)	35.0 (1.1)	33.9 (0.90)
**EE**	4.91 (0.25)	6.75 (0.22)	6.85 (0.25)	6.99 (0.25)
**Ash**	5.87 (0.31)	7.60 (0.12)	7.65 (0.74)	7.94 (0.21)

1 Nutrients are expressed on a dry matter basis except for dry matter, which is expressed on an as-fed basis. Apparent intakes were calculated as the feed delivered multiplied by the analyzed chemical composition of the feed, less the orts mass multiplied by the analyzed chemical composition of the orts. These apparent intakes were then divided by total dry matter intake to give the apparent diet chemical composition consumed.

2 Composition is reported as the mean (SD) across animal-days.

3 NFC = 100 − (CP + NDF + EE + ash).

#### Feeding procedure

The daily refusal sampling and feed dispensing activities occurred when the cows went to the parlor for afternoon milking. Animals were fed daily starting at 1300 h using a Calan data ranger (American Calan Inc.). Prior to feeding, a sample of refusals was collected and the weight of refusals was determined by removing feed from the bunk using the data ranger. After the bunks were emptied, the TMR was dispensed into each bunk within approximately 2.5 kg of the cow’s target as-fed amount for the day, and the exact amount dispensed was recorded. The TMR provided to each individual animal started at 47.7 kg/d on the first day of the adaptation period (d −14 to 0) and was varied daily to target 2 to 5 kg of refusals on an as-fed basis. After delivering the TMR to each animal daily, the designated top dress was weighed on a digital scale (Defender 5000 XtremeW, model T51XW; Ohaus Corp.), recorded, and hand mixed into the top third of the TMR in the bunk. Cows were allowed access to their feed after the feeding activities were completed at approximately 1530 h. The feed remained available until refusal collection on the following day. Research personnel were not blinded to treatments.

#### Feed sampling and determination of diet chemical composition

As previously described, samples of refusals were collected daily. Cow composited refusal samples within period were used for feed analysis, as described in the *Feed Analysis* section below. In addition to refusal samples, one TMR sample was obtained per period. The commodities used for top dress represented a single delivered batch of feed, and one sample of each ingredient was obtained for analysis. Daily weights for feed delivered and refusals were used, with time-matched chemical composition of feeds and refusals, to calculate apparent intake of nutrients for each diet. Apparent intake was calculated as the measured nutrient composition of the feed provided multiplied by the mass of feed provided, less the measured nutrient composition of the refusals multiplied by the mass of refusals each day. Apparent intakes were calculated for each cow, for each nutrient, for each day of available data (i.e., each day with refusals). These apparent intakes were used to calculate the chemical composition of each ration, as consumed, by dividing the apparent intake of each nutrient by the dry matter intake. Because analytical errors in feed analysis can occasionally result in the sum of nutrient intakes exceeding the measured dry matter intake, all nutrient intakes were standardized to dry matter intake prior to being used to calculate chemical composition. The mean apparent dietary chemical composition across cow days was calculated and reported along with the standard deviation of apparent composition as the best available representation of diet chemical composition consumed by cows on each treatment.

### Feeding algorithms for assignment of individual precision feeding strategy

The experiment was split into two phases to enable training of the precision feeding algorithms (periods 1 to 4) and testing of the efficacy of those algorithms in a practical feeding demonstration (periods 5 to 9).

#### Training phase

In the training phase, the 24 Holstein cows were randomly assigned via random number generator in Excel to 1 of 3 treatments or a control diet in a replicated 4 × 4 Latin square so that each group consisted of 6 animals. The number of cows chosen was consistent with that used in prior published literature (Price et al., 2021; Souza et al., 2022). To confirm adequacy of our sample size, we conducted a post hoc power analysis using the pwr package in R (Champely, 2020). With the observed standard deviation for milk yield (1.3 kg) and an alpha of 0.05, the achieved power to detect a 2.3 kg/d difference was 0.82. For DMI (SD = 0.73 kg), the power to detect a 2.9 kg/d difference was 0.85. This suggests that the sample size was sufficient to detect the magnitude of treatment effects observed in this study. The training phase lasted for 36 d and consisted of 4, 9-d periods. Because “animal period” is the proper experimental unit in a Latin square experiment, the sample size for this study was 96 animal periods (24 cows × 4 periods) with 24 animal periods allocated to each of the 4 groups (6 cows × 4 periods). One cow was removed on d 2 of period 3 for stealing feed from other bunks. Her data from all previous days were still utilized in the analysis. During each period of the training phase, cows received one of three top dress treatments or the TMR treatment. Treatment order was varied to help avoid confounding or carryover effects. Treatments are as previously described.

#### Testing phase

Immediately following the algorithm training phase, the 24 cows were randomly reassigned to 1 of 3 feeding groups. The first group (serving as the control) represented the traditional management style and was fed the TMR *ad libitum* without top dress. During this testing period, the cows in this group received only the control diet and did not receive the other experimental diets. The other feeding groups were fed one of the four experimental rations (SBM, CG + SBM, GLT + SBM, or control) based on the recommendation of one of two precision feeding approaches tested. The approach used to assign individual cows to diets is described in the *Feeding Algorithms* section below. In cases where a cow was assigned to receive the control diet rather than a top dress, the intent was to evaluate the potential effect of increased TMR quantity alone as an individualized feeding strategy. However, because TMR was already fed ad libitum with refusals targeted at 2 to 5 kg (as-fed), the practical impact of this adjustment was minimal, primarily serving as a control for evaluating composition-based precision feeding approaches. As previously stated, both approaches were trained using the individual cow data generated during the training phase. The testing phase ran for 35 d, consisting of five periods lasting 7 d each. One cow was removed on d 7 of period 2 for stealing feed from other bunks. Her data from all previous days were still utilized in the analysis. All other experimental conditions and procedures remained the same as in the training phase.

### Feeding algorithms

Although our overall study objectives included evaluating DMI and milk components as response variables, the precision feeding algorithms themselves were developed exclusively using standardized MY and feed efficiency FE. DMI and milk component outcomes were assessed to understand the broader impacts of the algorithms, but they were not part of the algorithmic selection or ranking criteria.

Prior to use in deriving the feeding approaches, the MY and FE data for each animal were standardized using the 9 days of data collected during each training period plus the available data collected during the testing period. A value of 1 would reflect a day where that cow produced 1 SD above her average milk production, while a value of −2 would reflect a day where that cow produced 2 SD below her average milk production. This standardization was conducted to normalize the data and to allow for better equating of the different performance parameters. In the feeding approach descriptions, reference to the performance data, MY, and FE refer to these standardized values only.

Two algorithms (Mean and Slope, described in detail below) were developed using the individual cow data collected during the training phase, and these same data informed the initial decisions in the testing phase. This design choice was intentional to simulate a real-time, reinforcement learning context, where the system continuously updates its understanding of each cow’s performance and immediately applies this information. Unlike traditional predictive modeling approaches, the algorithms were not developed to generalize to independent future data or to forecast absolute performance metrics. Instead, they were designed to iteratively adjust supplementation to optimize within-cow responses during the experimental period. Consequently, classical predictive model fit statistics (e.g., *R*^2^, RMSE, AIC) were not calculated, as they are not appropriate measures for the purpose of these algorithms. Rather, the effectiveness of each algorithm was evaluated through the observed changes in cow performance during the testing phase. No formal model selection criteria were used since the focus was on comparing algorithm-driven supplementation strategies rather than selecting or optimizing predictive features.

#### Approach 1 (mean)

In keeping with the concept of Occam’s Razor, our first strategy applied the simplest method for generating feed recommendations that could be devised by our research team. Recommendations were based on the mean previous performance on each feedstuff. In this feeding approach, all available days of training and test data were collected for each individual animal and categorized according to feeding strategy. The MY and FE averages attained for each feeding strategy were summarized. Feeding strategies were then ranked individually to reflect MY and FE performance on each top dress. Rankings were structured such that higher rankings (4 vs. 1) were reflective of greater MY and greater milk produced per unit of feed. The rank achieved for MY and FE was then summed to create a single ranking index for each feed. An example of this approach is shown in Table 4. The feedstuff with the highest rank score within each period was selected as the recommended feeding approach for that period. In instances where there was a tie in ranking, the feedstuff with more days of data available was used as the recommended feedstuff. This balancing of MY and FE was intended to support selection of feedstuffs that would not depress feed intake in the short term as a strategy to enhance FE.

**Table 4.** Example of the feedstuff selection method of algorithm 1

Feeding approach	Theoretical MY, kg/d	Theoretical FE (MY/DMI)	Rank for MY	Rank for FE	Rank total	Feedstuff selected
**Control (TMR)**	38.4	1.75	1	4	5	
**SBM**	40.5	1.70	2	3	5	
**CG + SBM**	40.7	1.60	3	1	4	
**GLT + SBM**	40.8	1.64	4	2	6	X

#### Approach 2 (slope)

Because the mean performance of an animal on a feedstuff does not explain much about the performance trend occurring while feeding, our second algorithm focused on the slope of performance outcomes occurring over the period of the training and testing phase during which the animal was on that feeding strategy. The slope-based approach was hypothesized to be superior because it accounts for time trends that might reflect advancing lactation or short-term adaptation. Slopes were calculated individually for each animal and feedstuff for MY and FE using least-squares regression of the performance outcomes relative to experimental day. Slopes were then ranked by performance type to indicate the feedstuff causing the most positive change in MY or FE over each feeding period. These ranks were structured and summed as described in approach 1 and used to define the optimal feeding strategy used. The overall hypothesis driving these feeding approaches was that cows have a repeatable relative performance change when experiencing short-term feedstuff supplementation, and that the short-term change can be used to reflect future performance on that feeding strategy, despite the ongoing shifts associated with progression through lactation. The slope-based strategy in algorithm 2 should allow for better control over normal changes occurring due to progression through lactation, and thus success of algorithm 2 over algorithm 1 might be indicative of an important role of stage of lactation in dictating precision feeding approaches.

### Experimental procedures

#### Milking

Cows were milked twice daily at approximately 0100 h and 1300 h in a double-12 De Laval parallel parlor (Dairymen Specialties, Inc.). Individual cow MY and milk composition analyses were recorded at each milking via an inline Afimilk MPC milk meter (Afimilk Ltd) and an AfiLab milk analyzer (Afimilk Ltd), respectively. Energy-corrected milk was calculated as: ECM (kg/d) = kg of milk × [(38.3 × % fat × 10 + 24.2 × % true protein × 10 + 16.54 × % lactose × 10 + 20.7)/3,140] (Sjaunja et al., 1990). Upon leaving the parlor, cow BW was recorded by a walkover scale equipped with a radiofrequency identification reader (AfiWeigh; Afimilk Ltd). All MY, milk composition, and BW data recordings were stored in the AfiFarm dairy herd management software (Afimilk Ltd).

#### Feed sampling and nutrient analysis

Daily samples of approximately 500 g (as-fed basis) were collected from fresh TMR and individual cow refusals. Refusal samples were collected using the quartering method (ServiTech Laboratories, 2021). All samples were stored in plastic zip-top bags and frozen at −20 °C. Samples of TMR and individual cow refusals were pooled by period. Because only one delivery load of CG, GLT, and SBM (Big Spring Mill Inc.) was utilized, initial samples of these feedstuffs were collected only at the beginning of the experiment. Feed samples were dried for 48 h at 55 °C in a forced-air oven (Thermo Scientific Heratherm Advanced Protocol Ovens Model 51028115, Fisher Scientific) to determine DM percentage and were ground to pass through a 1 mm screen of a Wiley mill (Model 4, Thomas Scientific). All feed samples were then subjected to analysis of NDF, ADF, ADL, CP, ether extract (EE), and ash content. Concentrations of NDF and ADF were obtained via an Ankom200 Fiber Analyzer (Ankom Technology). The NDF analysis used sodium sulfite (Fisher Scientific) and α-amylase from *Bacillus licheniformis* (Ankom Technology). Acid detergent lignin concentrations were determined by subjecting the residues from the ADF analysis to agitation in 72% sulfuric acid on a rocking platform (Flask Dancer, Boekel Scientific) for 3 h. Total N content was obtained by combustion analysis (Vario El Cube CN analyzer (Elementar Americas Inc.) and CP concentration was calculated as N percentage × 6.25. Ether extract was analyzed using the Ankom XT10 (Ankom Technology) with petroleum ether following manufacturer recommendations. Ash content was determined through combustion in a muffle furnace (Sybron Thermolyne FA1730, Fisher Scientific) for 8 h at 500 °C.

### Statistical analysis

The response variables examined included DMI, MY, FE (MY/DMI), milk fat percentage, milk fat yield, milk protein percentage, milk protein yield, ECM, corrected FE (ECM/DMI), and BW. All data were analyzed as a mixed-effects model in R version 4.3.2 (R Core Team, 2023) using the lme4 package (Bates et al., 2015). Analysis of data from the training phase included top dress type, day, and the interaction between top dress and day as fixed effects. An effect representing the initial level of a response variable from the adaptation to the beginning of the training phase (-14 to 0 d) was also included. Animal and period were treated as random effects. Period was included as a random effect to account for time-related variability and to represent periods as random samples of possible experimental time frames. Residuals were checked using Q–Q plots and were determined to follow a normal distribution. Analysis of variance was performed on each model. Estimated marginal means were calculated and means comparisons were made based on contrasts between the treatment groups (TMR plus top dresses) and the control group (TMR). Analysis of data from the testing phase utilized feeding group (mean, slope, or control), top dress within feeding group, and the initial level of a response variable (from the adaptation to the training phase) as fixed effects. Animal and period were once again treated as random effects and ANOVA was performed for each model. Estimated marginal means were calculated and means comparisons were made based on contrasts between the algorithm feeding groups and the control feeding group, and also between the subgroups assigned to top dresses or the control within the feeding groups. Significance was declared at *P *< 0.05 and tendencies at 0.05 ≤ *P *< 0.10. Classical predictive model selection metrics were not applied to the feeding algorithms, as these approaches were not intended as predictive models but rather as real-time, within-animal adaptive strategies evaluated directly through production outcomes.

### Economic comparison analysis

The financial impacts of employing the precision feeding algorithms were assessed by using the revenue from the milk produced during the study and the actual feed expenses corresponding to the different feeding strategies. Feed costs ($/hd/d) were computed as total feed consumed (kg/d) times its respective cost ($/kg, as fed). Milk revenue ($/hd/d) was calculated by multiplying milk production (kg/d) by milk price ($/kg). Income over feed costs (**IOFC**; $/hd/d) was obtained by subtracting feed cost from milk revenue. The commodity feeds utilized as top dresses in this study were sourced from Big Spring Mill (Elliston, VA). Actual purchase costs for TMR ($0.156/kg), corn grain ($0.288/kg), corn gluten feed ($0.294/kg), and soybean meal ($0.437/kg) fed during the study were used for feed cost estimation. Milk prices were those paid by the Commonwealth of Virginia under an annually negotiated contract. Milk sale receipts ($0.346/kg) were used to estimate milk revenues. All prices used were from the year 2019. Feed costs, milk revenues, and IOFC were subjected to the same statistical analysis procedures as the response variables analyzed from the algorithm testing phase as described above.

## Results

### Training phase

Means and significance values of performance responses measured during the algorithm training phase are presented in Table 5. Dry matter intake responded to top dress treatment (*P *< 0.001), being lowest in the Control diet and progressively higher in cows fed SBM, CG+SBM, and highest in those fed GLT+SBM. Milk yield increased 2.3 kg on average on the 3 top dress treatments compared to the Control diet, but the response to each top dress was similar (*P *> 0.05). Feed efficiency decreased on the top dress treatments compared to the Control treatment (*P *< 0.001). Feed efficiency was increased on SBM compared to GLT (1.69 vs. 1.61), while feed efficiency on CG was similar to SBM and GLT. Milk fat content and milk fat yield were both decreased (*P *< 0.05) in cows consuming SBM compared to cows consuming TMR. Milk protein content, milk protein yield, and cow BW did not differ with top dress (*P *> 0.27).

**Table 5.** Mean performance responses of cows fed a basal diet of TMR with the addition of CG+SBM, GLT+SBM, or SBM top dress, or no top dress (control) during the algorithm training phase

	Treatments					Significance values		
Response	Control	CG + SBM	GLT + SBM	SBM	SEM	Treatment	Day	Treatment × day
**DMI, kg/d**	21.9^d^	24.8^b^	25.5^a^	23.8^c^	0.73	<0.001	<0.001	0.003
**MY, kg/d**	38.4^b^	40.7^a^	40.8^a^	40.5^a^	1.3	<0.001	0.002	0.67
**Milk protein, %**	2.89	2.84	2.86	2.81	0.088	0.54	0.99	0.32
**Milk protein yield, kg/d**	1.11	1.14	1.16	1.13	0.051	0.28	0.62	0.28
**Milk fat, %**	4.32^b^	4.29^ab^	4.17^ab^	4.08^a^	0.087	0.02	0.88	0.51
**Milk fat yield, kg/d**	1.81^b^	1.79^ab^	1.75^ab^	1.71^a^	0.047	0.03	0.90	0.56
**FE, MY/DMI**	1.80^a^	1.65^bc^	1.61^b^	1.69^c^	0.049	<0.001	<0.001	0.28
**BW, kg**	685	685	685	684	12	0.88	<0.001	0.59

a–d Means within a row with different superscripts differ (*P *< 0.05).

**Figure 1.**
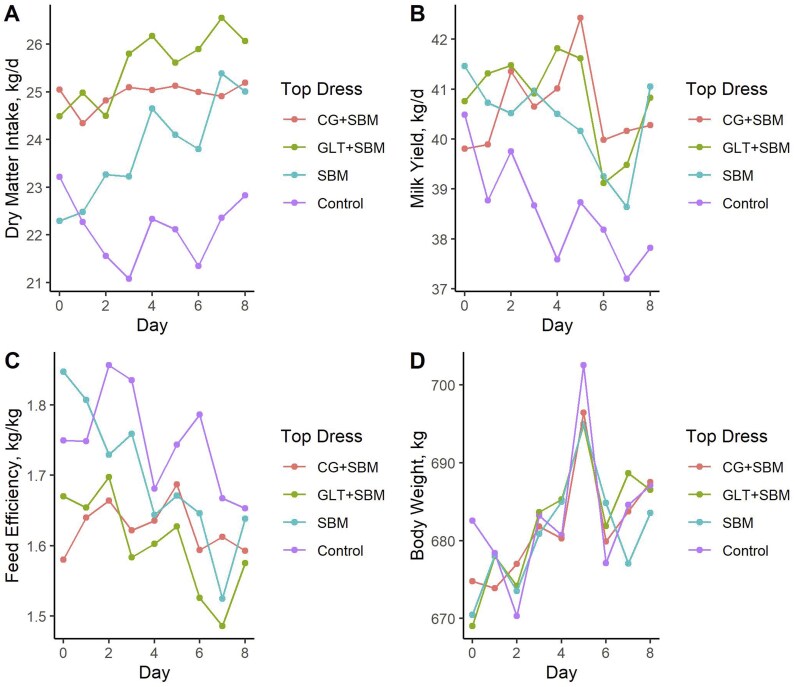
Change in performance responses over time during the algorithm training phase for (A) DMI, (B) milk yield, (C) feed efficiency, and (D) BW.

Four response variables of interest changed with respect to day (*P *< 0.003; Table 5) and are depicted in Figure 1. Although the observed variability in Figure 1 might raise concerns, our post hoc power analysis indicates that the sample size was sufficient to detect the treatment effects we observed for both milk yield and dry matter intake. Cow DMI increased over time with the exception of the Control diet (Figure 1A), while MY decreased (Figure 1B). Feed efficiency correspondingly decreased (Figure 1C). Body weight generally increased over time (Figure 1D). Milk components remained unaffected by time (Table 5). A treatment × day interaction was noted for DMI (*P *= 0.003; Table 5).

### Testing phase

Performance responses measured during the testing phase did not differ generally between the precision-fed groups and the traditionally fed control group (*P *> 0.06; Table 6). Dry matter intake tended to be higher in the precision-fed group using mean historical responses (feeding strategy 1) compared with the control group (*P *= 0.08; Table 6). Accordingly, the feed efficiency of the precision-fed group using mean historical responses tended to be lower than the control group (*P *= 0.07; Table 6). Performance responses of the 3 feeding groups as differentiated by top dress assignment are presented in Table 7, with their corresponding contrasts presented in Table 8. Cows consuming GLT in the mean-based approach had increased DMI compared to the control group (*P *= 0.03; Table 8), consuming 4.9 kg more on average (Table 7). They also exhibited poorer FE compared to the control group (*P *= 0.02; Table 8). Cows consuming CG in the mean-based approach tended to have increased DMI (*P *= 0.06; Table 8) and milk protein content (*P *= 0.09; Table 8) compared to the control-fed cows. No differences were observed between the cows precision-fed based on the slope of historical responses and the control group when top dress subgroup was considered (*P *> 0.12; Table 8). Irrespective of feeding approach, DMI, MY, FE, and corrected FE were significantly related to top dress type (*P *< 0.05; Table 7) and milk protein yield tended to be related to top dress type (*P *= 0.09). Milk fat percent, milk fat yield, BW, and ECM were unaffected by feeding group or top dress. With the exception of milk protein percent, all response variables of interest were affected by their initial values (*P *< 0.007; Table 7).

**Table 6.** Mean performance responses of cows fed during the algorithm testing phase using 1 of 2 precision feeding algorithms or a TMR control diet only, along with significance values for the contrasts

	Feeding group				Significance values	
Response	Control	Algorithm 1	Algorithm 2	SEM	Algorithm 1—Control	Algorithm 2—Control
**DMI, kg/d**	22.7	25.2	23.8	0.83	0.08	0.50
**MY, kg/d**	36.7	35.6	37.7	1.2	0.72	0.74
**Milk protein, %**	2.71	2.89	2.91	0.12	0.23	0.13
**Milk protein yield, kg/d**	1.03	1.02	1.09	0.05	>0.99	0.56
**Milk fat, %**	4.46	4.51	4.35	0.16	0.95	0.77
**Milk fat yield, kg/d**	1.83	1.90	1.80	0.07	0.74	0.87
**FE, MY/DMI**	1.61	1.45	1.58	0.05	0.07	0.89
**BW, kg**	677	678	681	5.4	0.96	0.79
**ECM, kg/d**	43.7	44.0	43.8	1.2	0.96	>0.99
**Corrected FE, ECM/DMI**	1.93	1.83	1.82	0.09	0.66	0.52

**Table 7.** Mean performance responses of cows fed during the algorithm testing phase using 1 of 2 precision feeding algorithms (broken down by top dress subgroup) or a TMR control diet only

	Treatments					Significance values		
Response	Control	CG + SBM	GLT + SBM	SBM	SEM	Initial value	Feeding group	Top dress
**DMI, kg/d**						<0.001	0.03	<0.001
**Algorithm 1**	22.6	26.3	27.6	24.2	1.1			
**algorithm 2**	21.7	24.6	25.0	24.0	0.94			
** Control**	22.7				0.86			
**MY, kg/d**						<0.001	0.75	<0.001
**Algorithm 1**	33.8	36.9	35.4	36.2	1.4			
**algorithm 2**	36.3	37.9	38.6	38.2	1.2			
**Control**	36.7				1.2			
**Milk protein, %**						0.22	0.05	0.32
**Algorithm 1**	2.83	3.07	2.91	2.76	0.16			
**algorithm 2**	3.10	2.87	2.84	2.81	0.14			
**Control**	2.71				0.11			
**Milk protein yield, kg/d**						<0.001	0.30	0.09
**Algorithm 1**	0.928	1.15	1.04	0.991	0.08			
**algorithm 2**	1.09	1.06	1.11	1.08	0.06			
**Control**	1.03				0.05			
**Milk fat, %**						0.001	0.73	0.53
**Algorithm 1**	4.6	4.56	4.45	4.43	0.29			
**algorithm 2**	4.75	4.36	4.09	4.18	0.24			
**Control**	4.46				0.17			
**Milk fat yield, kg/d**						<0.001	0.70	0.64
**Algorithm 1**	1.90	1.89	1.91	1.88	0.12			
**algorithm 2**	1.95	1.81	1.69	1.74	0.10			
**Control**	1.83				0.07			
**FE, MY/DMI**						0.006	0.17	0.02
**Algorithm 1**	1.53	1.45	1.29	1.51	0.07			
**algorithm 2**	1.66	1.55	1.54	1.59	0.06			
**Control**	1.61				0.06			
**BW, kg**						<0.001	0.49	0.25
**Algorithm 1**	674	678	693	668	7.4			
**Algorithm 2**	678	686	682	677	6.1			
**Control**	677				5.8			
**ECM, kg/d**						<0.001	0.34	0.73
**Algorithm 1**	42.8	46.0	43.2	44.1	2.1			
**Algorithm 2**	44.6	43.4	43.6	43.5	1.6			
**Control**	43.7				1.3			
**Corrected FE, ECM/DMI**						0.003	0.41	0.04
**Algorithm 1**	1.99	1.82	1.60	1.91	0.14			
**Algorithm 2**	2.06	1.76	1.69	1.77	0.11			
**Control**	1.93				0.09			

**Table 8.** Significance values for contrasts between the algorithm-fed groups of cows (broken down by top dress subgroup) and the control-fed group of cows on the basis of their productive responses

	Algorithm group 1—Control group				Algorithm group 2—Control group			
Response	Control	CG + SBM	GLT + SBM	SBM	Control	CG	GLT	SBM
**DMI, kg/d**	>0.99	0.06	0.03	0.77	0.93	0.52	0.27	0.81
**MY, kg/d**	0.42	>0.99	0.95	>0.99	>0.99	0.94	0.75	0.87
**Milk protein, %**	0.86	0.09	0.88	>0.99	0.13	0.67	0.79	0.94
**Milk protein yield, kg/d**	0.63	0.52	>0.99	>0.99	0.93	0.98	0.71	0.93
**Milk fat, %**	0.98	0.99	>0.99	>0.99	0.87	0.99	0.50	0.82
**Milk fat yield, kg/d**	0.96	0.96	0.99	>0.99	0.87	>0.99	0.64	0.89
**FE, MY/DMI**	0.79	0.31	0.02	0.75	0.97	0.93	0.84	>0.99
**BW, kg**	>0.99	>0.99	0.54	0.82	>0.99	0.77	0.94	>0.99
**ECM, kg/d**	0.99	0.71	>0.99	>0.99	0.99	>0.99	>0.99	>0.99
**Corrected FE, ECM/DMI**	0.98	0.92	0.47	>0.99	0.86	0.65	0.29	0.70

### Economic comparison

Feed costs, milk revenue, and IOFC did not differ generally between the precision-fed groups and the control group (*P *> 0.33; Table 9). Daily feed cost per head was numerically highest for cows in the precision feeding strategy based on historical means, particularly for those cattle consuming CG ($10.40; Table 10), but this only tended to be different from the control-fed group’s feed cost (*P *= 0.09; Table 11). Top dress type influenced feed costs and milk revenue (*P *< 0.003) and tended to influence IOFC (*P *= 0.06). All 3 variables were affected by their initial values (*P *< 0.02).

**Table 9.** Milk revenues and feed costs associated with cows fed during the algorithm testing phase using 1 of 2 precision feeding algorithms or a TMR control diet only, along with significance values for the contrasts

	Feeding group				Significance values	
Item^1^	Control	Algorithm 1	Algorithm 2	SEM	Algorithm 1—Control	Algorithm 2—Control
**Feed cost, $/hd/d**	9.07	9.31	9.70	0.33	0.34	0.82
**Milk revenue, $/hd/d**	12.4	12.6	13.0	0.55	0.94	0.60
**IOFC, $/hd/d**	3.33	2.84	3.71	0.49	0.70	0.80

1 IOFC (milk revenue − feed cost).

**Table 10.** Milk revenues and feed costs associated with cows fed during the algorithm testing phase using 1 of 2 precision feeding algorithms (broken down by top dress subgroup) or a TMR control diet only

	Treatment							Significance values		
Item^1^	Control		CG + SBM	GLT + SBM		SBM	SEM	Initial value	Feeding group	Top dress
**Feed cost, $/hd/d**								0.004	0.04	<0.001
** Algorithm 1**	9.08		10.4	9.97		9.32	0.42			
** Algorithm 2**	8.75		9.86	9.02		9.59	0.36			
** TMR control**	9.07						0.34			
**Milk revenue, $/hd/d**								<0.001	0.52	0.002
** Algorithm 1**	12.0		13.2	12.7		12.3	0.60			
** Algorithm 2**	12.5		13.1	13.2		13.4	0.56			
** TMR control**	12.4						0.57			
**IOFC, $/hd/d**								0.01	0.83	0.06
** Algorithm 1**	2.84		2.88	2.63		3.00	0.59			
** Algorithm 2**	3.69		3.18	4.20		3.77	0.51			
** TMR control**	3.33						0.51			

1 IOFC (milk revenue − feed cost).

**Table 11.** Significance values for contrasts between the algorithm-fed groups of cows (broken down by top dress subgroup) and the control-fed group of cows on the basis of milk revenues and feed costs

	Algorithm group 1—Control group				Algorithm group 2—Control group			
Item	Control	CG + SBM	GLT + SBM	SBM	Control	CG + SBM	GLT + SBM	SBM
**Feed cost, $/hd/d**	>0.99	0.09	0.58	0.98	0.96	0.49	>0.99	0.81
**Milk revenue, $/hd/d**	0.98	0.78	>0.99	>0.99	>0.99	0.87	0.80	0.69
**^1^IOFC, $/hd/d**	0.94	0.96	0.91	0.99	0.98	>0.99	0.70	0.96

1 IOFC (milk revenue − feed cost).

## Discussion

The design of this investigation allowed for the examination of the adaptive changes to individual cow productivity in response to the short-term provision of different concentrate feeds based on historical performance trends. This variation, both at the individual and group level, is often not captured in most traditional nutrition studies due to the utilization of dedicated adaptation periods and washout periods. Although these experimental approaches are vital in traditional hypothesis testing, they are impractical to apply in precision feeding contexts, meaning that examining continuous performance responses to short-term feeding interventions is necessary for the development of precision feeding approaches, which was a primary goal in this study.

It is important to emphasize that the provision of top-dress supplements in this study was not intended as a recommendation to feed nutrients in excess of requirements. Rather, the supplemental top-dresses served as an experimental tool to uncover variability in individual cow responses, acknowledging that even when a basal diet is formulated to meet the average requirements of the group, some cows may remain over- or under-supplied. This variability is precisely what precision or individualized feeding seeks to address, by tailoring nutrient supply to each cow’s specific needs or responses (Souza et al., 2022). In this context, the top-dress treatments in our study were used to reveal individual performance responses, rather than as a strategy to increase concentrate inclusion uniformly. Despite the expectation that these feeds would generate diets differing in RDP content with similar energy values (Table 2), the apparent composition of diets consumed was quite similar among treatments, with slight differences in CP, NFC, EE, and ash contents relative to the control (Table 3). Although diets were formulated to target a metabolizable energy intake of approximately 57 to 58 Mcal/d, the actual realized ME intake from the TMR was slightly lower (∼55 Mcal/d). This discrepancy reflects that cows consumed less feed than expected during the ration formulation process, resulting in lower realized ME intake despite alignment of dietary ME concentration with formulation targets. More drastic changes in diet may generate more realized shifts in feed efficiency; however, the top dress inclusion levels selected in this study were chosen, in part, to approximate a practical delivery ceiling for automated or robotic precision feeding systems, ensuring detectable and meaningful production responses without compromising cow health or intake behavior.

The algorithms trained using data generated from the first phase of this work were intended to optimize cow performance and were tested against a conventional, non-individualized feeding strategy. Although production responses were largely similar between the feeding strategies, these findings provide relevant information for the continued improvement of individualized feeding efforts. Considering that this design reflects the real-world application of reinforcement learning systems, the intent was not to create a generalizable feeding system, but rather to evaluate how well these algorithms could learn the responses of individual cows and shift those responses toward improved productivity. Thus, the study focused on illustrating some practical limitations of applying a reinforcement learning approach in a small example set of individual cows, rather than aiming for broad generalization.

### Training phase: production responses to changes in top dress

The increase in DMI observed in cows receiving top dress treatments is somewhat consistent with our previous observations, in which cows receiving the CG treatment consumed more feed than those consuming only TMR (Price et al., 2021). Increased DMI on ground corn grain has also been reported by Boerman et al. (2015). The increase in DMI on CG was expected, particularly given that our cows were around 130 DIM, a stage when rumen distension predominates as the primary regulator of intake, and hypophagic effects of starch are less pronounced (Allen, 2023). The inclusion of cracked corn may have reduced the effective fiber proportion and potentially decreased rumen fill effects and/or passage rate, allowing for increased DMI. Higher DMI associated with the SBM top dress compared to TMR is consistent with the well-established positive correlation between dietary CP content and intake (Allen, 2000; Zanton, 2016). Our observation of higher intakes in response to GLT treatment is also consistent with previous investigations on the effects of including corn gluten feed in lactating dairy cow diets (Sullivan et al., 2012; Hao et al., 2017). Although palatability likely played a role across all top dresses, an important additional factor is the substitution effect: as top dresses (CG, SBM, or GLT) replaced portions of the TMR, the dietary forage fiber content decreased while rapidly fermentable starch, protein, and non-forage fiber sources increased. This reduction in effective fiber content likely decreased rumen fill, increased passage rate, and promoted higher intake (Allen, 2000). The higher rate of fermentation would have accelerated nutrient availability, supporting greater milk production. Thus, the combined effects of altered rumen fill dynamics, enhanced fermentation, and palatability together explain the observed increases in DMI and MY across top dress treatments in our study. Collectively, these observations highlight the sensitivity of DMI to dietary ingredient composition, particularly in response to reduced forage fiber, increased passage rate, and enhanced fermentation dynamics resulting from concentrate top dresses.

Top dress effects on MY were consistent with our previous report focused on different precision feeding approaches and top dresses (Price et al., 2021), with higher yields in cows receiving concentrate top dresses than cows consuming the control TMR. This MY response indicates the inclusion of top dress treatments supported short-term production responses. The ability of supplementation, specifically of energy and protein sources, to augment milk production has been extensively documented in the literature (Coulon et al., 1987; Broderick, 2003; Benchaar et al., 2013; McKay et al., 2019; Doran et al., 2021). The increase in DMI observed with top dress treatments likely helped sustain these higher yields, given the well-established relationship between intake and milk production (Gleason and White, 2018). It is important to note, however, that at approximately 130 DIM, a stage when cows have typically passed peak MY and are at or near peak DMI. At this stage, increased intake primarily serves to support ongoing production and replenish body reserves, rather than directly driving further increases in milk yield (Ingvartsen and Andersen, 2000). Additionally, the decline in FE observed with top dress feeding suggests that the increase in MY did not occur through improved efficiency. This reduction in efficiency highlights the need to balance increases in absolute productivity and the growing demand for inputs, as well as the potential environmental and economic trade-offs. Feed efficiency was higher in cows assigned to the SBM treatment compared to GLT, but this appears to simply be a function of decreased DMI in the SBM-fed cows compared to the GLT-fed cows with no significant change in MY between the groups. The differences among top dresses demonstrate that milk production and feed efficiency may respond to abrupt changes in supplement provision, but that these changes are largely driven by shifts in DMI, further highlighting the significance of animal feeding behavior in driving productive output (Johnston and DeVries, 2018).

The decline in milk fat percentage observed with the SBM top dress, but not with CG or GLT, suggests that the mechanism may extend beyond a simple reduction in peNDF. Milk fat depression is often associated with low ruminal pH and altered biohydrogenation pathways resulting from increased intake of unsaturated fatty acids (Bauman and Griinari, 2003). Although SBM is not as lipid-rich as oilseeds, it does supply PUFA, and when combined with higher fermentability, may have promoted lower ruminal pH and shifts in fatty acid biohydrogenation. In contrast, CG and GLT top dresses included SBM but also provided additional starch or digestible fiber sources, potentially resulting in a more balanced supply of ruminal energy and protein. This could have supported rumen microbial activity and fiber digestion, helping to maintain a more stable rumen environment and mitigate milk fat depression (NASEM, 2021). Additionally, the partial substitution of forage with CG or GLT might have reduced peNDF content but did not appear to cause detrimental shifts in rumen fermentation, as evidenced by the absence of milk fat depression. It is important to clarify that CG and GLT top dresses in this study were provided as combinations including SBM, which may have further contributed to the observed responses.

Unlike milk fat, milk protein percentage and yield were not different across treatments. This was slightly surprising given that milk protein content has been demonstrated to increase linearly with dietary CP concentration (Katongole and Yan, 2020), and the inclusion of GLT and SBM would have raised. The base TMR, however, was formulated to meet or exceed cow nutrient requirements and apparent CP content consumed by cows was 16.7%. It is therefore possible that the lack of response in milk protein reflects the fact that cows were not protein-deficient on the TMR, and additional increases in dietary protein did not translate into further improvements in milk protein yield because the mammary gland was no longer limited by amino acid supply (NASEM, 2021). Consequently, the mammary cellular machinery likely had sufficient precursors for protein synthesis, explaining the similar treatment responses observed here.

Cow BW was not significantly affected by treatment, which was expected due to the short timespan spent on each treatment (9 d). In addition, although DMI changed in response to top dress, the differences in DMI between the groups were likely not extreme enough to result in noticeable variation in BW, at least over the amount of time dedicated to evaluation. Our observation is consistent with that of Reynal and Broderick (2003), who reported no change in cow BW gain on various concentrate supplements over 21 d despite significant differences in DMI. Although we did not examine BCS as a response variable in this study, our cows were apparently not losing or accruing body fat to any significant degree in response to treatment.

Across the 9-day treatment periods, DMI, MY, FE, and BW varied with respect to day, with DMI generally increasing in cows receiving the top dress treatments but remaining variable in the TMR control group, MY and FE declining, and BW increasing. These changes may be partly explained by gradual adaptation of the rumen microbial population to the new dietary components, which typically stabilizes within 6 to 13 days following a diet change (Machado et al., 2016). Cows receiving the top dresses appeared to respond to the dietary disruption by steadily increasing their intakes the longer the same diet was provided. The treatment × time interaction noted for DMI appeared to be due to a decline in intake of the TMR-fed cows during the first half of the period followed by an unsteady recovery, but a cause for this decline was not immediately apparent.

Because days in milk only advanced by approximately 9 d within each treatment period, the general decline in MY for all groups over time is likely due to cumulative effects of the experimental treatments and day-to-day intake dynamics rather than true lactation stage effects. This short duration is insufficient to cause significant metabolic changes typically associated with lactation progression (Harder et al., 2019). The more extreme reduction in MY exhibited by the TMR-fed cows was most likely due to the earlier decline in DMI (Hristov et al., 2005). The decline in FE over time is logical given the general increase in DMI and the concurrent decrease in MY. The observed gradual increase in BW across periods may reflect modest energy surplus from increased intake rather than a true lactation stage recovery effect. Overall, the short treatment periods and day-to-day intake variations are more likely to explain these observed production changes than small shifts in DIM alone.

### Testing phase: evaluation of precision feeding performance

Despite our efforts to develop feeding algorithms for the optimization of MY and FE, cow performance was virtually unchanged whether cows were precision-fed or traditionally-fed. Cows fed by approach 1 (based on mean historical responses) tended to have increased DMI and decreased FE compared to the traditionally-fed cows while cows fed by approach 2 (based on the slope of historical responses) performed similarly to the control group in all variables measured. The absence of a significant algorithm effect on milk components is probably not unexpected, given that the approaches in this study were intended to specifically target MY and efficiency responses rather than the optimization of individual components. These feeding approaches represent somewhat of an improvement upon previous algorithm development efforts (Souza et al., 2022) in that they avoided targeting depressed feed intake as a way to drive short-term improvements in FE. The lack of positive improvement in present cow performances, however, suggests that the new algorithms overcorrected and instead relied too heavily on DMI to attempt to drive increases in MY such that FE was ultimately not improved.

The differences in performance between cows fed based on mean historical responses and the control group appear to be top dress-specific, with precision-fed cows receiving GLT exhibiting increased DMI and decreased FE. This is consistent with the effect of GLT treatment observed during the training phase. The tendency of cows fed CG based on mean historical responses to consume more feed than the control-fed cows was also in agreement with our prior observations. The tendency of increased milk protein percent in these same cows compared to the control-fed group possibly occurred if the CG increased microbial protein production in the rumen, which may have supplied more AA to the mammary gland. Further investigation would be required to determine if an effect exists in this case and if this potential mode of action was responsible. Top dress impacted MY similarly to the algorithm training phase, but interestingly was not identified as a significant factor for milk fat content or yield, indicating that milk fat may not be as consistently responsive to top dress supplementation. The lack of top dress effect on BW, however, remained consistent between the training and testing phases.

As explained above, feeding approaches were either based on mean historical performance or feeding based on the slopes of production responses over the course of supplementation. Cows that were fed based on mean responses tended to have increased DMI and decreased FE compared to the traditionally-fed cows. However, cows fed based on the rate of change of responses performed similarly to the traditionally-fed group. Therefore, our original expectation that feeding based on the slope of the response would outperform feeding based on mean response was somewhat correct. This suggests that considering production trends over time in response to dietary shifts may have merit over solely examining mean production values on differing feedstuffs, particularly given that cow production parameters would be expected to shift over a normal lactation cycle (Angeles-Hernandez et al., 2021; Seymour et al., 2021).

### Economic analysis of feeding approaches

The slightly increased feed cost associated with the feeding based on mean historical performance is logical, given the higher mean DMI observed in this group; however, this did not result in a significant difference in feed costs compared to the traditionally fed group. As would be anticipated given the lack of significant difference in milk production responses between the algorithm-fed and traditionally-fed cows, the feed costs incurred and milk revenues generated during this feeding period were also not different. Ultimately, this financial examination indicated no remarkable cost or benefit associated with the use of the precision feeding strategies compared to the Control diet (conventional TMR strategy).

### Limitations and future directions

Despite the promising insights from this study, several important limitations should be acknowledged, and future directions considered. First, the supplementation strategy relied on partial top-dress substitutions rather than fully rebalanced individualized rations, which may have led to subtle nutrient imbalances and variable cow adaptation responses. This may have contributed to the variability and limited ability of the algorithms to learn from the past responses. Finally, it should be noted that partial replacement of TMR with top dress treatments also reduced the absolute dietary supply of feed additives included in the base TMR (e.g., palmitic acid, monensin, yeast, Zn), as these were not re-supplemented in the top dress formulations. Whether this contributed to some of the differences observed in production and efficiency responses remains unclear.

During the evaluation of top dress treatment effects prior to algorithm testing, no treatments resulted in enhanced FE. Milk yield was elevated on average in cows receiving top dress provision compared to the TMR control (Table 5). However, within each 9-d treatment period, MY tended to decline over time in all groups (Figure 1B), indicating that the higher overall yield did not prevent the expected short-term declining trend during each period. This was also coupled with a sufficient increase in DMI such that FE was poorer for all supplementation strategies compared to no supplementation. It is therefore possible that adjusting dietary energy and protein substrate types alone may not be the most effective strategy to enhance overall performance. Feed restriction has not generally been viewed as an optimal strategy to increase efficiency since it is usually accompanied by a substantial decline in milk production (Ben Meir et al., 2019; Herve et al., 2019). However, Fischer et al. (2020) demonstrated that targeted feed restriction using a precision feeding approach could improve FE and reduce methane emissions without compromising MY or body condition, by setting individualized intake limits for the least efficient cows rather than restricting the entire group uniformly. While the approach in our study focused on increasing nutrient supply through top dress supplementation, the findings of Fischer and colleagues highlight an alternative strategy for individual-level nutritional management aimed at improving efficiency. This contrast suggests that future work could explore both precision supplementation and precision restriction strategies, depending on the target production or environmental outcomes.

Despite tending to result in better efficiency outcomes than feeding based on historical mean performance, feeding based on the slope of performance changes was unable to outperform the conventional feeding strategy for any production parameter considered. This suggests that animal production responses to supplement provision did not necessarily remain consistent when past performance or performance trends were used to inform future supplementation decisions. The same observation was made previously (Souza et al., 2022), and suggests that the development of precision feeding strategies that can 1) be implemented in practical production settings and 2) result in consistent and reliable performance optimization for individuals is likely to be very complex. Advancement in this area may perhaps be achieved by employing machine learning techniques, which can be informed by data collected on individual animals through precision technologies (Kaur et al., 2023). Campos et al. (2023) developed a mathematical framework for the optimization of precision-fed dairy cattle diets using different feeding approaches and an objective function set to optimize milk income minus feed cost. Their simulations indicate lower-cost diets ($0.17 cow/d), greater milk (0.4 L/cow/d), slightly greater milk income ($0.16 cow/d), and increased milk income minus feed cost ($0.32 cow/d) for cows receiving individualized grain mixes as compared to those that were fed with clustered solutions. Although their system is promising, it requires the application of their optimized solutions on farms to calculate returns and compare them with optimized predictions. Other potential avenues may involve simulation studies or leveraging the Molly cow model (Li et al., 2018); however, these options do not provide reflections of individual animals. Regardless of precisely how future decision-making surrounding successful precision feeding strategies may be achieved, it will require a greatly improved understanding of individual animal performance variation and how the productive potential therein can be effectively harnessed in a commercial setting.

Another important consideration is the use of the same historical dataset for both algorithm development and testing phases, which inherently limits external validation and generalizability. This design reflects the real-world application of reinforcement learning systems, which rely on immediate feedback from individual cows to refine feeding strategies in an ongoing manner. However, it also implies that the algorithms were not validated on truly independent data, which may constrain the robustness of conclusions if extrapolated to different herds or management settings. Future research should explore the development of individualized, fully balanced rations tailored to each cow’s real-time nutrient requirements and consider strategies that integrate both supplementation and controlled restriction to optimize performance and efficiency. Incorporating additional data streams, such as real-time rumen fermentation or metabolic markers, may also improve algorithm accuracy and reliability, potentially enhancing the reliability of precision feeding approaches.

In addition to the experimental and algorithmic considerations discussed above, certain practical limitations related to data collection and sensor reliability should also be noted. One such limitation is the absence of records regarding the calibration frequency of the AfiLab sensor during the data collection period, as well as the lack of DHIA test results for comparison. Although milk composition data were not used in the algorithm training or evaluation and thus did not affect model performance, we acknowledge that sensor-based milk composition data can be prone to error if not regularly calibrated. Future studies should ensure routine calibration of in-line sensors and consider validating sensor outputs against standardized laboratory tests, such as DHIA, to enhance data reliability.

## Conclusions

Lactating dairy cows may respond to individualized feeding of supplemental energy or protein sources by exhibiting shifts in their production parameters. However, approaches designed to provide feeding recommendations on the basis of these responses were unable to optimize future milk production or feed efficiency compared to a traditionally-fed, un-supplemented TMR. Past performance response to a dietary intervention therefore may not always be a reliable prediction of future performance response to the same intervention, at least for an individual animal. Future efforts to develop improved precision feeding strategies may need to identify alternative approaches to predicting the performance behavior of individual cows.

## Data Availability

The data and R codes that support the findings of this study are available from the corresponding author upon reasonable request. *Conflict of interest statement.* The authors declare that the research was conducted in the absence of any commercial or financial relationships that could be construed as a potential conflict of interest.

## Abbreviations:

ADL: acid detergent lignin
CG: corn grain
FE: feed efficiency
GLT: corn gluten feed
IOFC: income over feed costs
MHA: methionine hydroxy analogue
MP: metabolizable protein
MY: milk yield
NEm: net energy for maintenance
peNDF: physically effective NDF
RUP: rumen undegradable protein
SBM: soybean meal

## References

[skaf317-B1] Allen M. S. 2000. Effects of diet on short-term regulation of feed intake by lactating dairy cattle. J. Dairy Sci. 83:1598–1624. 10.3168/jds.S0022-0302(00)75030-2 10908065

[skaf317-B2] Allen M. S. 2023. Symposium review: integrating the control of energy intake and partitioning into ration formulation. J. Dairy Sci. 106:2181–2190. 10.3168/jds.2022-22473 36631325

[skaf317-B3] Andreen D. M. , Salfer I. J., Ying Y., Reinemann D. J., Harvatine K. J. 2020. Technical note: method for improving precision of in-parlor milk meters and adjusting milk weights for stall effects. J. Dairy Sci. 103:5162–5169. 10.3168/jds.2019-17479 32307171

[skaf317-B4] Angeles-Hernandez J. C. , Aranda-Aguirre E., Muñoz-Benítez A. L., Chay-Canul A. J., Albarran-Portillo B., Pollott G. E., Gonzalez-Ronquillo M. 2021. Physiology of milk production and modelling of the lactation curve. CABI Rev. 60:1–22. 10.1079/pavsnnr202116056

[skaf317-B5] Bates D. , Mächler M., Bolker B., Walker S. 2015. Fitting linear mixed-effects models using lme4. J. Stat. Softw. 67:1–48. 10.18637/jss.v067.i01

[skaf317-B6] Bauman D. E. , Griinari J. M. 2003. Nutritional regulation of milk fat synthesis. Annu. Rev. Nutr. 23:203–227. 10.1146/annurev.nutr.23.011702.073408 12626693

[skaf317-B7] Beauchemin K. A. 2018. Invited review: current perspectives on eating and rumination activity in dairy cows. J. Dairy Sci. 101:4762–4784. 10.3168/jds.2017-13706 29627250

[skaf317-B8] Ben Meir Y. A. , Nikbachat M., Portnik Y., Jacoby S., Levit H., Bikel D., Adin G., Moallem U., Miron J., Mabjeesh S. J., et al 2019. Dietary restriction improved feed efficiency of inefficient lactating cows. J. Dairy Sci. 102:8898–8906. 10.3168/jds.2019-16321 31351720

[skaf317-B9] Benchaar C. , Hassanat F., Gervais R., Chouinard P. Y., Julien C., Petit H. V., Massé D. I. 2013. Effects of increasing amounts of corn dried distillers grains with solubles in dairy cow diets on methane production, ruminal fermentation, digestion, N balance, and milk production. J. Dairy Sci. 96:2413–2427. 10.3168/jds.2012-6037 23462175

[skaf317-B10] Boerman J. P. , Potts S. B., VandeHaar M. J., Allen M. S., Lock A. L. 2015. Milk production responses to a change in dietary starch concentration vary by production level in dairy cattle. J. Dairy Sci. 98:4698–4706. 10.3168/jds.2014-8999 25981075

[skaf317-B11] Broderick G. A. 2003. Effects of varying dietary protein and energy levels on the production of lactating dairy cows. J. Dairy Sci. 86:1370–1381. 10.3168/jds.S0022-0302(03)73721-7 12741562

[skaf317-B12] Campos L. M. , Ringer H., Chung M., Hanigan M. D. 2023. Application of a mathematical framework for the optimization of precision-fed dairy cattle diets. Animal 17:101001. 10.1016/j.animal.2023.101001 39492077

[skaf317-B13] Champely S. 2020. pwr: Basic Functions for Power Analysis. Version 1.3-0., Vienna, Austria.

[skaf317-B14] Connor E. E. 2015. Invited review: improving feed efficiency in dairy production: challenges and possibilities. Animal 9:395–408. 10.1017/S1751731114002997 25482927

[skaf317-B15] Coulon J. B. , Petit M., D’Hour P., Garel J. P. 1987. The effect of level and distribution of concentrate supplementation on performance of dairy cows. Livest. Prod. Sci. 17:117–133. 10.1016/0301-6226(87)90058-3

[skaf317-B16] da Rosa Righi R. , Goldschmidt G., Kunst R., Deon C., André da Costa C. 2020. Towards combining data prediction and internet of things to manage milk production on dairy cows. Comput. Electron. Agric. 169:105156. 10.1016/j.compag.2019.105156

[skaf317-B17] Doran M. J. , Mulligan F. J., Lynch M. B., O’Sullivan M., Fahey A. G., McKay Z. C., Brady E. L., Grace C., O’Rourke M., Pierce K. M. 2021. Effects of genotype and concentrate supplementation on milk composition and selected milk processability parameters in late-lactation spring-calving grazing dairy cows. Int. Dairy J. 114:104942. 10.1016/j.idairyj.2020.104942

[skaf317-B18] Fischer A. , Edouard N., Faverdin P. 2020. Precision feed restriction improves feed and milk efficiencies and reduces methane emissions of less efficient lactating Holstein cows without impairing their performance. J. Dairy Sci. 103:4408–4422. 10.3168/jds.2019-17654 32113758

[skaf317-B19] Gleason C. B. , White R. R. 2018. Variation in animal performance explained by the rumen microbiome or by diet composition. J. Anim. Sci. 96:4658–4673. 10.1093/jas/sky332 30124869 PMC6247856

[skaf317-B20] Hao X. Y. , Gao H., Wang X. Y., Zhang G. N., Zhang Y. G. 2017. Replacing alfalfa hay with dry corn gluten feed and Chinese wild rye grass: effects on rumen fermentation, rumen microbial protein synthesis, and lactation performance in lactating dairy cows. J. Dairy Sci. 100:2672–2681. 10.3168/jds.2016-11645 28215882

[skaf317-B21] Harder I. , Stamer E., Junge W., Thaller G. 2019. Lactation curves and model evaluation for feed intake and energy balance in dairy cows. J. Dairy Sci. 102:7204–7216. 10.3168/jds.2018-15300 31202643

[skaf317-B22] Herve L. , Quesnel H., Veron M., Portanguen J., Gross J. J., Bruckmaier R. M., Boutinaud M. 2019. Milk yield loss in response to feed restriction is associated with mammary epithelial cell exfoliation in dairy cows. J. Dairy Sci. 102:2670–2685. 10.3168/jds.2018-15398 30639009

[skaf317-B23] Hristov A. N. , Price W. J., Shafii B. 2005. A meta-analysis on the relationship between intake of nutrients and body weight with milk volume and milk protein yield in dairy cows. J. Dairy Sci. 88:2860–2869. 10.3168/jds.S0022-0302(05)72967-2 16027201

[skaf317-B24] Ingvartsen K. L. , Andersen J. B. 2000. Integration of metabolism and intake regulation: a review focusing on periparturient animals. J. Dairy Sci. 83:1573–1597. 10.3168/jds.S0022-0302(00)75029-6 10908064

[skaf317-B25] Johnston C. , DeVries T. J. 2018. Short communication: associations of feeding behavior and milk production in dairy cows. J. Dairy Sci. 101:3367–3373. 10.3168/jds.2017-13743 29397173

[skaf317-B26] Katongole C. B. , Yan T. 2020. Effect of varying dietary crude protein level on feed intake, nutrient digestibility, milk production, and nitrogen use efficiency by lactating Holstein-Friesian cows. Animals (Basel) 10:2439. 10.3390/ani10122439 PMC7766052 33352790

[skaf317-B27] Kaur U. , Malacco V. M. R., Bai H., Price T. P., Datta A., Xin L., Sen S., Nawrocki R. A., Chiu G., Sundaram S., et al 2023. Invited review: integration of technologies and systems for precision animal agriculture-a case study on precision dairy farming. J. Anim Sci. 101:1–23. 10.1093/jas/skad206 PMC10370899 37335911

[skaf317-B28] Li M. M. , White R. R., Hanigan M. D. 2018. An evaluation of Molly cow model predictions of ruminal metabolism and nutrient digestion for dairy and beef diets. J. Dairy Sci. 101:9747–9767. 10.3168/jds.2017-14182 30243626

[skaf317-B29] Machado M. G. , Detmann E., Mantovani H. C., Valadares Filho S. C., Bento C. B. P., Marcondes M. I., Assunção A. S. 2016. Evaluation of the length of adaptation period for changeover and crossover nutritional experiments with cattle fed tropical forage-based diets. Anim. Feed Sci. Technol. 222:132–148. 10.1016/j.anifeedsci.2016.10.009

[skaf317-B30] Mayo L. M. , Silvia W. J., Ray D. L., Jones B. W., Stone A. E., Tsai I. C., Clark J. D., Bewley J. M., Heersche G. 2019. Automated estrous detection using multiple commercial precision dairy monitoring technologies in synchronized dairy cows. J. Dairy Sci. 102:2645–2656. 10.3168/jds.2018-14738 30692002

[skaf317-B31] McKay Z. C. , Lynch M. B., Mulligan F. J., Rajauria G., Miller C., Pierce K. M. 2019. The effect of concentrate supplementation type on milk production, dry matter intake, rumen fermentation, and nitrogen excretion in late-lactation, spring-calving grazing dairy cows. J. Dairy Sci. 102:5042–5053. 10.3168/jds.2018-15796 30981482

[skaf317-B32] NASEM. 2021. Nutrient requirements of dairy cattle: eighth revised edition. The National Academies Press, Washington, DC. 38386771

[skaf317-B33] NRC. 2001. Nutrient requirements of dairy cattle, 7th rev. ed. National Academy Press, Washington, D.C.

[skaf317-B34] Price T. P. , Souza V. C., Liebe D. M., Elett M. D., Davis T. C., Gleason C. B., Daniels K. M., White R. R. 2021. Short-term adaptation of dairy cattle production parameters to individualized changes in dietary top dress. Animals 11:3518. 10.3390/ani11123518 34944293 PMC8697869

[skaf317-B35] R Core Team. 2023. A language and environment for statistical computing. R Found. Stat. Comput., Vienna, Austria.

[skaf317-B36] Reynal S. M. , Broderick G. A. 2003. Effects of feeding dairy cows protein supplements of varying ruminal degradability. J. Dairy Sci. 86:835–843. 10.3168/jds.S0022-0302(03)73666-2 12703620

[skaf317-B37] Roelofs J. B. , van Eerdenburg F. J. C. M., Soede N. M., Kemp B. 2005. Pedometer readings for estrous detection and as predictor for time of ovulation in dairy cattle. Theriogenology 64:1690–1703. 10.1016/j.theriogenology.2005.04.004 15904954

[skaf317-B38] ServiTech Laboratories. 2021. Feed Sampling Procedures. https://servitech.com/laboratory-services/feed-and-forage-testing/feed-sampling-procedures (accessed January 30, 2021).

[skaf317-B39] Seymour D. J. , Cánovas A., Chud T. C. S., Cant J. P., Osborne V. R., Baes C. F., Schenkel F. S., Miglior F. 2021. Associations between feed efficiency and aspects of lactation curves in primiparous Holstein dairy cattle. J. Dairy Sci. 104:9304–9315. 10.3168/jds.2020-20010 33934862

[skaf317-B40] Sjaunja L. O. , Baevre L., Junkkarinen L., Pedersen J., Setälä J. 1990. A Nordic proposal for an energy corrected milk (ECM) formula. In: Proceedings of the 27th Biennial Session of the International Committee for Animal Recording (ICAR), Paris, France. p. 156–157.

[skaf317-B42] Souza V. C. , Liebe D. M., Price T. P., Ellett M. D., Davis T. C., Gleason C. B., Daniels K. M., White R. R. 2022. Algorithm development for individualized precision feeding of supplemental top dresses to influence feed efficiency of dairy cattle. J. Dairy Sci. 105:4048–4063. 10.3168/jds.2021-20841 35248384

[skaf317-B43] Sullivan M. L. , Grigsby K. N., Bradford B. J. 2012. Effects of wet corn gluten feed on ruminal pH and productivity of lactating dairy cattle fed diets with sufficient physically effective fiber. J. Dairy Sci. 95:5213–5220. 10.3168/jds.2012-5320 22916927

[skaf317-B44] Zanton G. I. 2016. Analysis of production responses to changing crude protein levels in lactating dairy cow diets when evaluated in continuous or change-over experimental designs. J. Dairy Sci. 99:4398–4410. 10.3168/jds.2015-10438 27060818

